# Identification of neuropeptides and neuropeptide receptor genes in *Phauda flammans* (Walker)

**DOI:** 10.1038/s41598-022-13590-7

**Published:** 2022-06-14

**Authors:** Hai-Pan Wu, Xiao-Yun Wang, Jin Hu, Ran-Ran Su, Wen Lu, Xia-Lin Zheng

**Affiliations:** Guangxi Key Laboratory of Agric-Environment and Agric-Products Safety, National Demonstration Center for Experimental Plant Science Education, College of Agriculture, Guangxi University, Nanning, 530004 China

**Keywords:** Entomology, Transcriptomics

## Abstract

Neuropeptides and neuropeptide receptors are crucial regulators to insect physiological processes. The 21.0 Gb bases were obtained from Illumina sequencing of two libraries representing the female and male heads of *Phauda flammans* (Walker) (Lepidoptera: Phaudidae), which is a diurnal defoliator of ficus plants and usually outbreaks in the south and south-east Asia, to identify differentially expressed genes, neuropeptides and neuropeptide receptor whose tissue expressions were also evaluated. In total, 99,386 unigenes were obtained, in which 156 up-regulated and 61 down-regulated genes were detected. Fifteen neuropeptides (i.e., F1b, Ast, NP1, IMF, Y, BbA1, CAP2b, NPLP1, SIF, CCH2, NP28, NP3, PDP3, ARF2 and SNPF) and 66 neuropeptide receptor genes (e.g., A2-1, FRL2, A32-1, A32-2, FRL3, etc.) were identified and well-clustered with other lepidopteron*.* This is the first sequencing, identification neuropeptides and neuropeptide receptor genes from *P. flammans* which provides valuable information regarding the molecular basis of *P. flammans*.

## Introduction

Insect neuropeptides as a classic signaling molecule are produced by the neurosecretory cells that are mainly located in the brain and the central nervous system, among others, to reach their distant target organs^1^. They are small proteins with generally about 5–80 amino acid residues, which are one of the structurally most diverse signaling molecules and most diverse group of signaling molecules in multicellular organisms^2,3^. Most neuropeptide receptors belong to the family of G protein-coupled receptor (GPCR), and most of the neuropeptides act via G protein coupled receptors^4,5^. It has been widely reported that neuropeptide and their receptors participate in intercellular information transfer from neurotransmission to intrinsic or extrinsic neuromodulation and essential signaling molecules that regulate physiological processes such as growth, development, behavior, reproduction, metabolism and muscle movement in insects^2–4,6,7^.

For now, a plethora of neuropeptides and receptors were investigated in insects, such as myoinhibiting peptides (MIPs)^8–14^, and so forth. Among these, PBAN, galanin and melanocortin are involved in the control of reproduction^10,15^. NPY is regulating feeding and sleep–wake behavior^16^. Thus, neuropeptides and their receptors could be developed as potential insecticides or targets for a novel generation of pesticides^17^, such as the neuropeptide CCH was proved to be regulates feeding motivation and sensory perception and olfactory behavior^18,19^ and the enteroendocrine peptides allatotropin (AT) and GSRYamide have feeding acceleratory effects via controlling intestinal contraction^20^. Therefore, identification and functional characterization of neuropeptides and their receptors from insect pests would enhance our basic understanding of neuropeptide-related signal transduction, and provide important molecular insights for pest management. Up to now, neuropeptide and receptors have been the focus of interest in many species of Lepidoptera, such as *Manduca sexta*^21–24^, which are mainly nocturnal moths. While, few researches have been reported on diurnal moth of Lepidoptera except for silkworm and butterfly^25,26^.

The diurnal moth *Phauda flammans* (Walker) (Lepidoptera: Phaudidae) is a serious defoliator which intermittent outbreaks that threaten ficus plant seriously, especially *Ficus microcarpa* (Miq.) and *F. benjamina* L.^27^. It not only influences the urban landscapes and ecological effects, but also affects normal growth and development of ficus plant^28–31^. This defoliator is abundantly distributed in south and south-east Asia and southern China^32^. At present, most of the researches about *P. flammans* focus on its morphological characteristics^33–40^. However, the research on neuropeptides and their receptors in *P. flammans* has been limited in comparison to other lepidopteran insects, due to lack of availability of genomic or transcriptomic information.

In this study, we conducted high-throughput sequencing of head, identified members of the neuropeptide and neuropeptide receptor of *P. flammans,* and compared them with those reported transcriptome of other lepidopteran species for the first time. We also evaluated the expression level of 12 neuropeptides in different adult tissues. Our results could provide useful information of neuropeptide and their receptor and theoretical basis for their functional analysis.

## Materials and methods

### Insect rearing and tissue collection for RNA-seq

The mature larvae of *P. flammans* were collected from July to October 2020 in Daxin County (22°50′10 N, 107°12′27E), Chongzuo City, Guangxi Province, China, and placed in plastic boxes (diameter = 25.0 cm, height = 15.0 cm) that supplied with fresh ficus leaves per day, at an indoor temperature with 26 ± 2 ℃, 70 ± 10% relative humidity (RH) with a photoperiod cycle of 14 h L/10 h D. Differentiate male and female pupae according to their ventral segments and randomly select 1-day-old healthy male and female adults for the experiment after feathering. The tissues head from adult male (n = 90) and female (n = 90) were collected. All samples were immediately frozen in liquid nitrogen and stored at − 80 °C until use.

### RNA-seq

Total RNA of *P. flammans* was extracted by TRIzol (Thermo Fisher Scientific, Waltham, MA) following the manufacturer’s instructions. The integrity of the RNA was determined with an Agilent 2100 bioanalyzer through agarose gel electrophoresis. The Nanodrop micro-spectrophotometer (Thermo Fisher, USA) was determined the purity and concentration of the RNA. After total RNA extraction, transcriptome sequencing was performed on an Illumina NovaSeq 6000 by Gene Denovo Biotechnology Co. (Guangzhou, China). To obtain high quality clean reads, reads were further filtered with fastp (version 0.18.0), mainly by removing reads containing adapters, removing reads containing more than 10% unknown nucleotides (N), and removing low quality reads with > 50% low quality reads (*q* value ≤ 20). Reads were then mapped to the ribosomal RNA (rRNA) database using the short reads matching tool Bowtie2 (version 2.2.8). The mapped rRNA reads were removed, and the remaining clean reads were assembled by the short read assembly program Trinity v3.030 to obtain the total unigene. The transcriptomic data were submitted to the National Center for Biotechnology Information (NCBI, USA) (http://www.ncbi.nlm.nih.gov/) with accession number of PRJNA702893.

### Transcriptome data analysis

The unigene expression was calculated and normalized to RPKM (Reads Per kb per Million reads)^41^ and the relative expression of differential expressed genes were viewed by volcano plot.

Unigene sequences were aligned by BLASTx and TBLASTx searches against the protein database (http://blast.ncbi.nlm.nih.gov/) such as NCBI non-redundant protein (Nr) database, SwissProt database, KEGG Ontholog database (KO) and Gene Ontology (GO) for annotation information. The transcriptomic (RNA-seq) data derived from *P. flammans* were used for identification of the neuropeptides and receptors.

### Sequence analysis and phylogenetic tree analysis

Transmembrane domains (TMDs) were calculated using the TMHMM 2.0 prediction software (http://www.cbs.dtu.dk/services/TMHMM/). The presence of signal peptide was predicted using SignalP software version 4.1 (http://www.cbs.dtu.dk/services/SignalP/). The splice sites were predicted using the Known Motif and Insect Models methods of NeuroPred (http://stagbeetle.animal.uiuc.edu/cgi-bin/neuropred.py) and were corrected based on the processing procedures of known insect neuropeptide precursors. Thesequence alignments were done using CLUSTALW, the result were implemented in MEGAv7.034 and GeneDoc software. With tBLASTn, the available sequences proteins from lepidoptera species were used as queries to identify candidate unigene involved in neuropeptides and neuropeptide receptor genes in *P. flammans*. To construct an evolutionary tree of neuropeptides and receptors, the amino acid sequences of the *Atrijuglans hetaohei*, *Bombyx mori*, *Chilo suppressalis*, *H. armigera*, *Grapholita molesta*, *Ostrinia furnacalis, Papilio machaon* and *Pl. xylostella* were downloaded from the NCBI database and performed in MEGA7 and the tree was constructed using the Neighbor-Joining method with 1000 bootstraps.

### Tissue expression profile via quantitative PCR

The head (without antennae), thoraxes (without legs), abdomens were dissected from 15 virgin 1-day-old of females or males, respectively. These tissues were immediately transferred into 1.5 mL RNA-free tube, super-cooled via liquid nitrogen, and then stored at − 80 °C freezer. These tissues were used for RNA extraction with RNAiso Plus (TAKARA, 9109, Dalian, China) and then cDNA synthesis with A Prime Script RT reagent Kit with gDNA Eraser (TAKARA, RR047, Dalian, China). The quantitative PCR reactions were conducted on an ABI QuantStudioTM 6 Flex system (Thermo Fisher Scientific, Massachusetts, USA). The PCR reaction was performed with each reaction was performed with Green Premix Ex Taq II Kit (TAKARA, RR820A, Dalian, China) and prepared as introduced^42^. The expression level of target gene was normalized with reference gene *TUB1* (α-tubulin) and *GAPDH* (*Glyceraldehyde-3-phosphate dehydrogenase*) via 2^-∆∆CT^ method according to our previous works^39,42^. The primers used in this research were listed in the Table S1.

### Statistical analysis

The normality and homoscedasticity of data on tissue expression of neuropeptides in female and male *P. flammans* adults were tested prior to analysis using Kolmogorov–Smirnov and Levene’s tests, respectively. And, they were further analyzed using one-way analysis of variance (ANOVA) followed by Tukey’s honestly significant difference (HSD) multiple test (*P* < 0.05). Data analysis was performed using SPSS 25.0 (IBM Corp., Armonk, New York, USA).

## Results

### Overview of cephalic transcriptomes

The cDNA libraries were constructed from *P. flammans* tissue samples of male and female heads to next-generation sequencing analysis by using Illumina HiSeq (TM) 4000 platform. A total of 21.0 G of clean bases were obtained, Q20 and Q30 values were all > 93%, and GC content was 39.82 ~ 40.87%. The combined unigene of *P. flammans* were functionally annotated by BLASTx according to six functional public databases: NCBI non-redundant protein (Nr), the Kyoto Encyclopedia of Genes and Genomes (KEGG) database, Swiss-Prot, Cluster of Orthologous Groups (COG) and gene ontology (GO) (e value < 0.00001). A total of 99,386 unigene (average length 911 bp) were obtained with 37,602, 28,494, 17,458, 19,910 annotations to the Nr, KEGG, KOG, SwissProt databases, respectively. A total of 40,131 annotations, account for 40.38% of the total unigene (Table 1).

**Table 1:** The four major databases annotate the statistics of *P. flammans*.

Details	Number
Clean reads from all samples (Gb)	21.00
Q20 (%)	97.72 ~ 97.93
Q30 (%)	93.18 ~ 93.00
GC content (%)	39.82 ~ 40.87
Total unigene	99,386
Average length of total unigene (bp)	911
N50 of unigene (nt)	11,923
Unigene with homolog in Nr	37,602
Unigene with homolog in KEGG	28,494
Unigene with homolog in Swiss-Prot	19,910
Unigene with homolog in KOG	17,458
Total number of annotation genes	40,131

**Figure 1:**
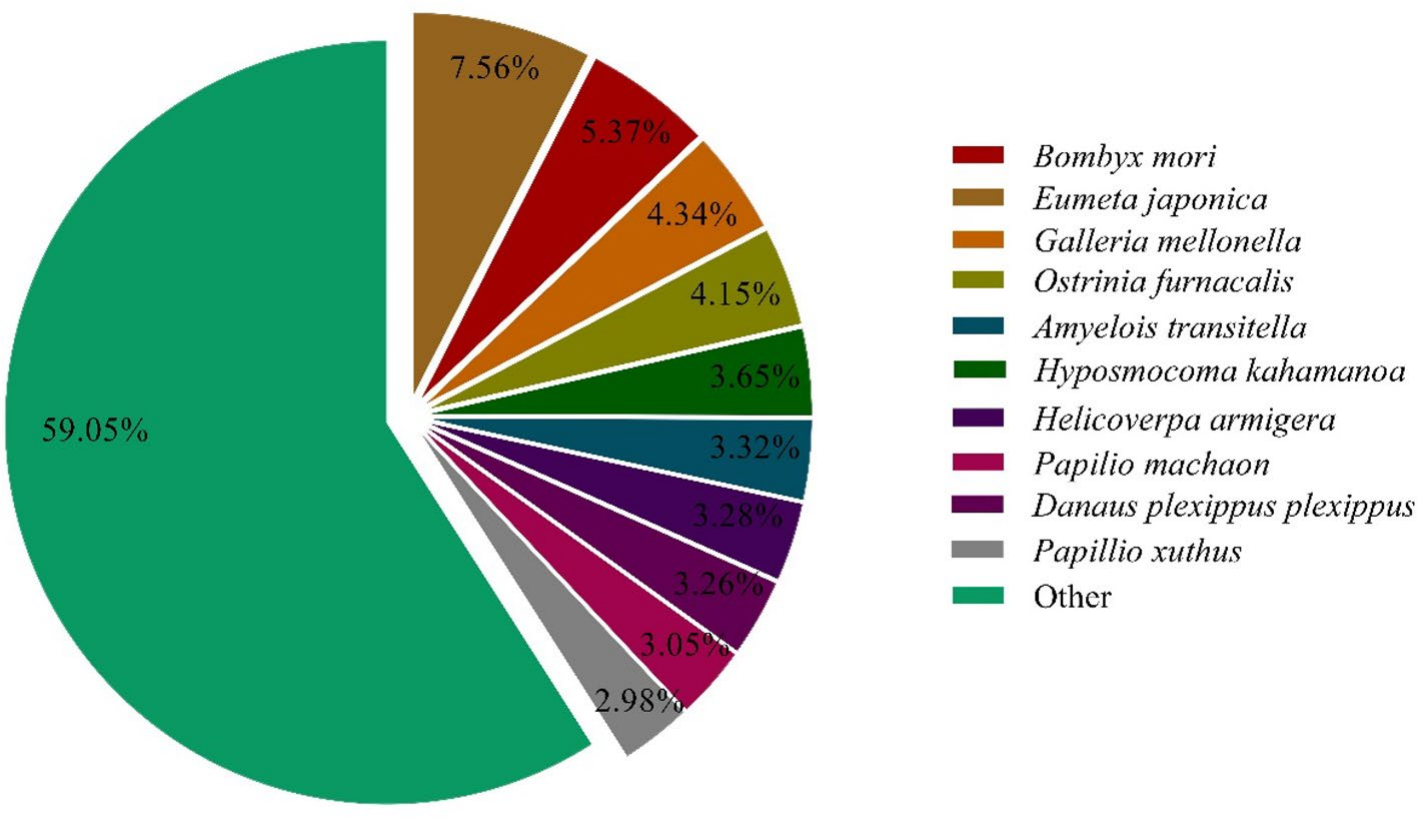
Species distribution based on Nr alignment results of head transcriptome of *P. flammans* unigene.

The Nr databases comprise all non-redundant protein sequences in GenBank, EMBL, DDBJ and PDB that belong to phylogenies of more than 70,000 species. Based on Nr annotation, unigene sequences of *P. flammans* can be mapped with sequences from 10 top species (Fig. 1). The number of homologous sequences sorted from most to least is *Eumeta japonica* (7.56%), *B. mori* (5.37%), *Galleria mellonella* (4.34%), *O. furnacalis* (4.15%), *Hyposmocoma kahamanoa* (3.65%), *Amyelois transitella* (3.32%), *H. armigera* (3.28%), *Danaus plexippus* (3.26%), *Pa. machaon* (3.05%), and *Pa. xuthus* (2.98%).

### Differentially expressed genes (DEGs) between female and male heads

**Figure 2:**
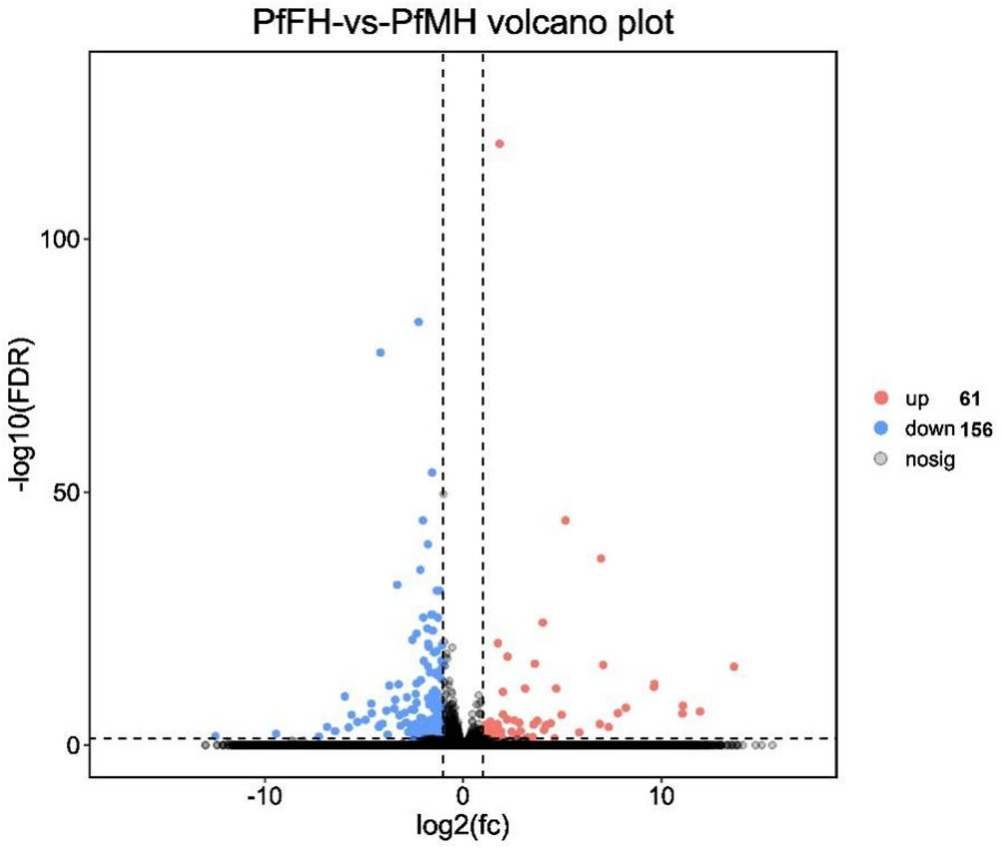
Volcano plot of differentially expressed genes in FH and MH head of *P. flammans.*

The results of differential expression analysis of genes in the heads of male and female adult *P. flammans* showed that a total of 217 differentially expressed genes were screened, with 156 genes up-regulated and 61 genes down-regulated, using FDR < 0.05 and |log2FC|> 1 as screening criteria (Fig. 2). The detailed information about these DGEs were listed in the supporting information 1.

### Identification of neuropeptides and their receptors

The neuropeptides in *P. flammans* were identified (Table 2). The neuropeptides F1b, Ast, NP1, IMF, Y, BbA1, CAP2b, NPLP1, SIF, CCH2, NP28, NP3, PDP3, ARF2, and SNPF were identified from the data sets with the length between 331 and 2947 bp. Except for NP1 and BbA1 have 3′ non-coding regions, and the others had complete open reading coding frames (ORFs), including F1b, Ast, IMP, Y, NP2, NPLP1, SIF, CCH2, NPLP28, NPLP3, PDP3, ARF2 and SNPF. Fifteen neuropeptides except for ARNPFT2 had signal peptide, and their signal peptide most likely cleavage site between 16 to 28.

**Table 2:** Neuropeptide identified in *P. flammans*.

Gene Name	Unigene ID	Unigene length (bp)	ORF (aa)	Complete ORF	SP (aa)	Homology search with known protein				
						Name	Species	E-value	Accession No	Identity (%)
Neuropeptide Ast	Unigene0011781	1111	125	YES	28	Allatostatin neuropeptide	*Operophtera brumata*	3e-39	KOB78759	66.67
Neuropeptide ARF2	Unigene0098383	2620	194	YES	NO	Antho-RFamide neuropeptide type 2	*Folsomia candida*	1e-28	OXA46921	39.63
Neuropeptide BbA1	Unigene0062770	331	90	NO	20	Neuropeptide precursor ILB1	*Plodia interpunctella*	6e-08	QDO72232	40.20
Neuropeptide CAP2b	Unigene0064787	1091	176	YES	19	Insect neuropeptide	*Chilo suppressalis*	7e-49	ALM30308	50.28
Neuropeptide CCH2	Unigene0077813	2947	132	YES	23	Neuropeptide CCHamide-2	*Zerene cessonia*	5e-37	XP_038220225	62.70
Neuropeptide F1b	Unigene0007256	754	121	YES	22	Neuropeptide F1b	*Grapholita molesta*	4e-65	QMS43307	80.17
Neuropeptide IMF	Unigene0020806	848	76	YES	28	Neuropeptide IMFamide	*Trichoplusia ni*	7e-41	XP_026736927	73.40
Neuropeptide NP1	Unigene0018659	1491	308	NO	23	PREDICTED: LWamide neuropeptides	*Papilio machaon*	9e-99	XP_014363816	64.18
Neuropeptide NPLP1	Unigene0069233	2299	462	YES	26	Neuropeptide-like 1	*Papilio machaon*	0.0	KPJ13870	62.50
Neuropeptide NP3	Unigene0088075	506	91	YES	16	Neuropeptide-like 3	*Ostrinia furnacalis*	3e-05	XP_028167263	78.79
Neuropeptide NP28	Unigene0086051	454	128	YES	21	Neuropeptide-like protein 28	*Trichoplusia ni*	0.0	XP_026744361	47.83
Neuropeptide PDP3	Unigene0097416	1234	166	YES	17	Putative defense protein 3	*Galleria mellonella*	6e-95	XP_026755410	81.93
Neuropeptide SNPF	Unigene0099186	1070	178	YES	27	Short neuropeptide F	*Bombyx mandarina*	3e-60	XP_028027333	67.96
Neuropeptide SIF	Unigene0075624	838	75	YES	22	Neuropeptide SIFamide	*Helicoverpa armigera*	1e-20	AGH25569	60.29
Neuropeptide Y	Unigene0024892	550	94	YES	21	Neuropeptide Y	*Helicoverpa assulta*	1e-37	AEE01342	82.98

SP: signal peptide; NO: no signal peptide; N: most likely cleavage site. ORF: open reading frame.

**Figure 3:**
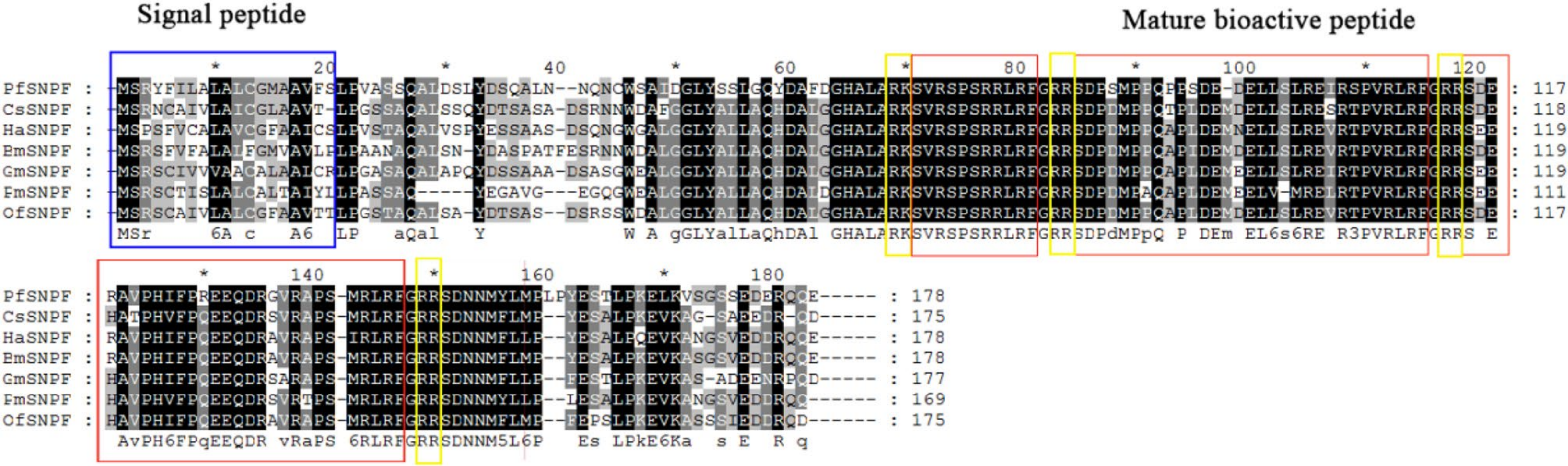
Multiple alignment of amino acid sequences of SNPF precursors from several lepidopteran. The blue box indicates the signal peptides; the red box indicates the sequences of mature bioactive neuropeptides; the yellow box indicates the dibasic cleavage sites.

The PfSNPF precursor had an N-terminal signal peptide of 19 amino acids and 3 mature SNPF were generated by sulfidation modifications. The PfSNPF precursor contained the –RLRF sequence, which belongs to the C-terminal motif unique to the SNPF family. Thereafter followed an amidation site (G) and a dibasic cleavage site (RR). The multiple alignments also showed that the SNPF of *P. flammans* had a higher similarity with other lepidopteron (Fig. 3).

Of the data sets, 67 neuropeptide receptors were identified (Table 3). Thirty-six neuropeptide receptors have completed ORFs. A2-1, FRL2, A32-1, A32-2, FRL3, CCH1R-1, B1-2 FRL5, A10-1, CPR3, A21-2, FRL6, A19, RYES2L1, RYES2L2, A16, CC1R1 and CRLIX2 comprise 3′ non-coding region. Versus those sequences were published such as *A. hetaohei*, *B. mori*, *C. suppressalis* and *H. armigera*, the number of neuropeptides in *P. flammans* is much lower than those in other insects.

**Table 3:** Neuropeptide receptors identified in *P. flammans*.

Gene name	Unigene ID	ORF (aa)	Complete ORF	Homology search with known protein				
				Name	Species	E-value	Accession No	Identity (%)
A1	Unigene0064405	415	YES	Neuropeptide receptor A1	*Chilo suppressalis*	0.0	ALM88296.1	82.09
A2-1	Unigene0016063	147	NO	Neuropeptide FF receptor 1-like isoform X2	*Danaus plexippus plexippus*	2e-75	XP_032511519.1	85.33
A2-2	Unigene0019915	340	YES	Neuropeptide receptor A2	*Chilo suppressalis*	4e-147	ALM88297.1	64.54
A5	Unigene0083958	538	YES	Neuropeptide receptor A5	*Chilo suppressalis*	0.0	ALM88300.1	84.31
A6	Unigene0055636	562	YES	Neuropeptide receptor A6-B	*Chilo suppressalis*	0.0	ALM88302.1	81.64
A7	Unigene0024096	444	YES	Neuropeptide receptor A7	*Bombyx mori*	0.0	NP_001127724.1	81.11
A8	Unigene0040364	431	YES	Neuropeptide receptor A8 isoform X2	*Bombyx mori*	0	XP_021205802.1	81.02
A10-1	Unigene0034954	49	NO	Neuropeptide receptor A10	*Chilo suppressalis*	2e-12	ALM88306.1	62.26
A10-2	Unigene0018800	434	YES	Neuropeptide receptor A10	*Chilo suppressalis*	0.0	ALM88306.1	82.05
A11	Unigene0000113	379	YES	Neuropeptide receptor A11	*Chilo suppressalis*	0.0	ALM88307.1	76.20
A12	Unigene0059896	218	YES	Neuropeptide FF receptor 2-like	*Bicyclus anynana*	1e-101	XP_023954589.1	76.47
A13	Unigene0036770	394	YES	Neuropeptide receptor A13	*Chilo suppressalis*	0	ALM88309.1	87.87
A14	Unigene0085759	411	YES	Neuropeptide receptor A14	*Chilo suppressalis*	0.0	ALM88310.1	87.72
A15	Unigene0097454	385	YES	Neuropeptide CCHamide-1 receptor-like	*Trichoplusia n*	0.0	XP_026732623.1	88.05
A16	Unigene0045083	55	NO	Neuropeptide receptor A16	*Chilo suppressalis*	9e-19	NP_001127740.1	69.64
A17	Unigene0053791	374	YES	Neuropeptide receptor A17	*Chilo suppressalis*	2e-162	ALM88313.1	81.90
A19-1	Unigene0040713	289	NO	Neuropeptide receptor A19	*Bombyx mori*	3e-124	NP_001127717.1	76.13
A19-2	Unigene0075821	127	YES	Neuropeptide receptor A19	*Danaus plexippus plexippus*	2e-29	OWR50546.1	54.33
A20	Unigene0050336	400	YES	Neuropeptide receptor A20	*Bombyx mori*	2e-171	NP_001127718.1	68.15
A21-1	Unigene0006521	431	YES	Neuropeptide receptor A21	*Chilo suppressalis*	0.0	ALM88307.1	72.25
A21-2	Unigene0035868	137	NO	Neuropeptide receptor A21	*Bombyx mori*	4e-64	NP_001127719.1	79.71
A21-3	Unigene0039820	99	YES	Neuropeptide receptor A21	*Chilo suppressalis*	1e-41	ALM88317.1	74.75
A21-4	Unigene0039821	73	YES	Neuropeptide receptor A21	*Chilo suppressalis*	1e-16	ALM88317.1	85.71
A23	Unigene0080116	492	YES	Neuropeptide receptor A23	*Chilo suppressalis*	0.0	ALM88319.1	78.03
A24	Unigene0019565	404	YES	Neuropeptide receptor A24 isoform X2	*Bombyx mori*	0.0	XP_021206901.1	72.06
A26	Unigene0021511	431	YES	Neuropeptide receptor A26	*Bombyx mori*	0.0	NP_001127724.1	74.69
A27-1	Unigene0022662	206	YES	Neuropeptide receptor A27	*Chilo suppressalis*	5e-63	ALM88323.1	76.92
A27-2	Unigene0071820	227	NO	Neuropeptide receptor A27	*Chilo suppressalis*	5e-104	ALM88323.1	89.43
A30	Unigene0050344	427	YES	Neuropeptide receptor A30	*Bombyx mori*	0.0	NP_001127746.1	78.92
A32-1	Unigene0025790	62	NO	Neuropeptide receptor A32	*Bombyx mori*	6e-22	NP_001127748.1	72.46
A32-2	Unigene0025792	54	NO	Neuropeptide receptor A32	*Chilo suppressalis*	1e-26	ALM88328.1	89.09
A33-1	Unigene0000680	56	YES	Neuropeptide receptor A33	*Operophtera brumata*	0.001	KOB79390.1	44.68
A33-2	Unigene0009036	285	YES	Neuropeptide receptor A33	*Bombyx mori*	1e-158	NP_001127749.1	84.86
A33-3	Unigene0009037	467	NO	Neuropeptide receptor A33	*Bombyx mori*	1e-14	NP_001127749.1	63.27
A33-4	Unigene0031688	153	YES	Neuropeptide receptor A33	*Operophtera brumata*	5e-04	KOB79390.1	41.33
B1-1	Unigene0096987	113	YES	Neuropeptide receptor B1	*Bombyx mori*	2e-28	NP_001127732.1	56.70
B1-2	Unigene0004595	142	YES	Neuropeptide receptor B1	*Chilo suppressalis*	4e-24	ALM88307.1	73.57
B3-1	Unigene0032437	99	NO	Neuropeptide receptor B1 receptor	*Grapholita molesta*	1e-49	QPZ46794.1	76.00
B3-2	Unigene0032438	36	YES	Neuropeptide receptor B3	*Operophtera brumata*	2e-04	KOB76486.1	61.11
B4	Unigene0046571	252	YES	Neuropeptide receptor B4	*Danaus plexippus plexippus*	3e-79	OWR44767.1	55.79
CC1R1	Unigene0049151	48	NO	Neuropeptide CCHamide-1 receptor	*Eumeta japonica*	5e-08	GBP60316.1	61.90
CC1R2	Unigene0067409	108	YES	Neuropeptide CCHamide-1 receptor	*Eumeta japonica*	2e-08	GBP60316.1	60.00
CCH1R-1	Unigene0032166	72	NO	Neuropeptide CCHamide-1 receptor	*Eumeta japonica*	2e-04	GBP60316.1	54.55
CPRLIX3	Unigene0087541	56	YES	Neuropeptides capa receptor-like isoform X3	*Ostrinia furnacalis*	9e-06	XP_028172560.1	77.42
CPRL	Unigene0062957	87	YES	LOW QUALITYES PROTEIN: neuropeptides capa receptor-like	*HYESposmocoma kahamanoa*	2e-14	XP_026317849.1	44.32
CPR1	Unigene0032922	72	YES	Neuropeptide capa receptor	*Folsomia candida*	3e-10	QXA62831.1	53.97
CPR2	Unigene0033466	94	YES	Neuropeptide capa receptor	*Folsomia candida*	6e-18	QXA62831.1	40.74
CPR3	Unigene0035545	62	NO	Neuropeptide capa receptor	*Neuropeptide capa receptor*	3e-08	QXA62831.1	55.74
CPR4	Unigene0059281	569	YES	Neuropeptides capa receptor	*Dufourea novaeangliae*	2e-72	KZC04374.1	47.33
CRL	Unigene0053966	190	YES	Neuropeptide capa receptor-like	*Papilio xuthus*	2e-43	XP_013175162.1	43.27
CRLIX2	Unigene0053965	83	NO	Neuropeptides capa receptor	*Papilio machaon*	4e-20	KPJ4363.1	56.47
LGR2	Unigene0040715	25	YES	PREDICTED: neuropeptide YES receptor-like	*AmYESelois transitella*	9e-05	XP_013200170.1	84.00
F	Unigene0070754	421	YES	Neuropeptide F receptor	*Danaus plexippus plexippus*	0.0	XP_032523519.1	86.35
FR1	Unigene0008530	146	YES	Neuropeptide F receptor-like	*Spodoptera litura*	8e-20	XP_022827582.1	52.38
FFR1	Unigene0016061	185	YES	Neuropeptide FF receptor 1-like	*Spodoptera litura*	2e-59	XP_022816539.1	75.94
FRL1	Unigene0024448	48	YES	Neuropeptide F receptor-like	*Spodoptera litura*	0.005	XP_022827582.1	47.92
FRL2	Unigene0013541	57	YES	Neuropeptide F receptor-like	*Spodoptera litura*	0.002	XP_022827582.1	60.00
FRL3	Unigene0024885	43	NO	Neuropeptide F receptor-like	*Spodoptera litura*	2e-09	XP_022827582.1	67.50
FRL4	Unigene0028572	136	NO	Neuropeptide F receptor-like	*Spodoptera litura*	6e-10	XP_022827582.1	50.00
FRL5	Unigene0031532	67	YES	Neuropeptide F receptor-like	*Spodoptera litura*	1e-08	XP_022827582.1	48.28
FRL6	Unigene0034532	199	NO	Neuropeptide F receptor-like	*Spodoptera litura*	4e-29	XP_022827582.1	57.73
FRL7	Unigene0036369	65	NO	Neuropeptide F receptor-like	*Spodoptera litura*	3e-08	XP_022827582.1	46.43
FRL8	Unigene0058443	54	NO	Neuropeptide F receptor-like	*Spodoptera litura*	4e-05	XP_022827582.1	41.82
RYES2L1	Unigene0041516	141	NO	Neuropeptide YES receptor tYESpe 2-like	*Trichoplusia ni*	1e-62	XP_026747410.1	73.64
RYES2L2	Unigene0043926	131	NO	Neuropeptide YES receptor tYESpe 2-like	*Trichoplusia ni*	2e-62	XP_026747410.1	73.64
SIFR	Unigene0078153	475	YES	PREDICTED: neuropeptide FF receptor 2-like	*Papilio polYEStes*	0.0	XP_013164121.1	87.79
YR2L	Unigene0070564	44	YES	PREDICTED: neuropeptide YES receptor tYESpe 2-like	*Diuraphis noxia*	2e-06	XP_015379364.1	52.27

### Phylogenetic analyses

**Figure 4:**
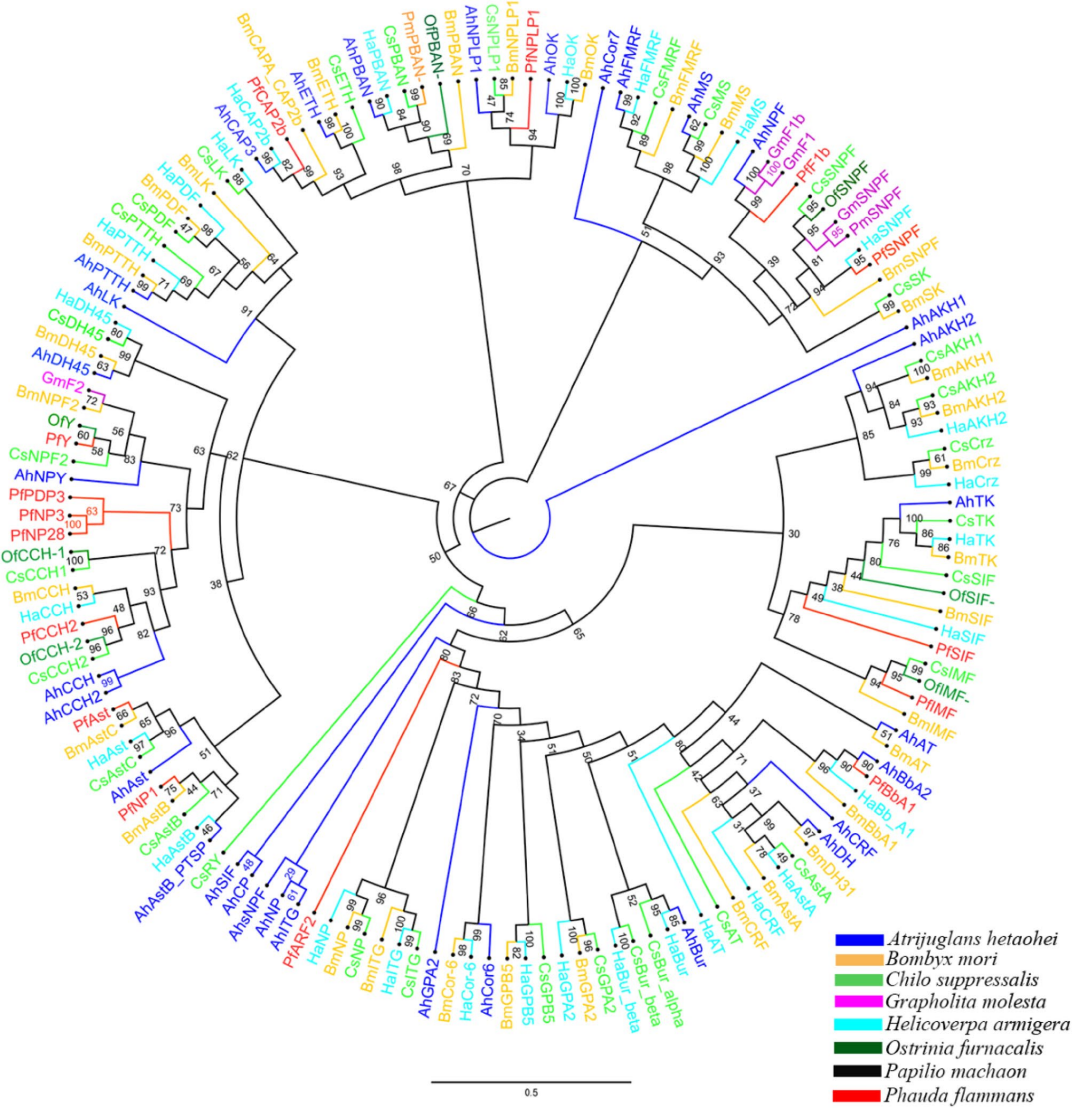
Phylogenetic analysis of lepidopterous neuropeptides. Ah: *A. hetaohei*; Bm: *B. mori*; Cs: *C. suppressalis*; Ha: *H. armigera*; Gm: *G. molesta*; Pf: *P. flammans*, Pm: *Pa. machaon*; Of: *O. furnacalis.* The *P. flammans* neuropeptide are labeled with red, and the colors of other species are shown in the icon. The tree was conducted with MEGA 7.0, using the Maximum-Likelihood method and the bootstrap analysis with 1000 replicates.

Neuropeptide sequences of *P. flammans* were used to construct maximum likelihood phylogenetic trees with 137 published neuropeptide sequences from lepidoptera including *A. hetaohei*, *B. mori*, *C. suppressalis*, *H. armigera*, *G. molesta*, *Pa. machaon* and *O. furnacalis* (Fig. 4). Among all neuropeptides, F1b, Ast, NP1, IMF, Y, BbA1, CAP2b, NPLP1, SIF, CCH2, NP28, NP3, PDP3, ARF2, and SNPF were clustered together with the orthologs from other lepidoptera insects in the same clade. On the contrary, ARF2 and NP28 in a single clade, they are considered as owning special function in the *P. flammans*.

**Figure 5:**
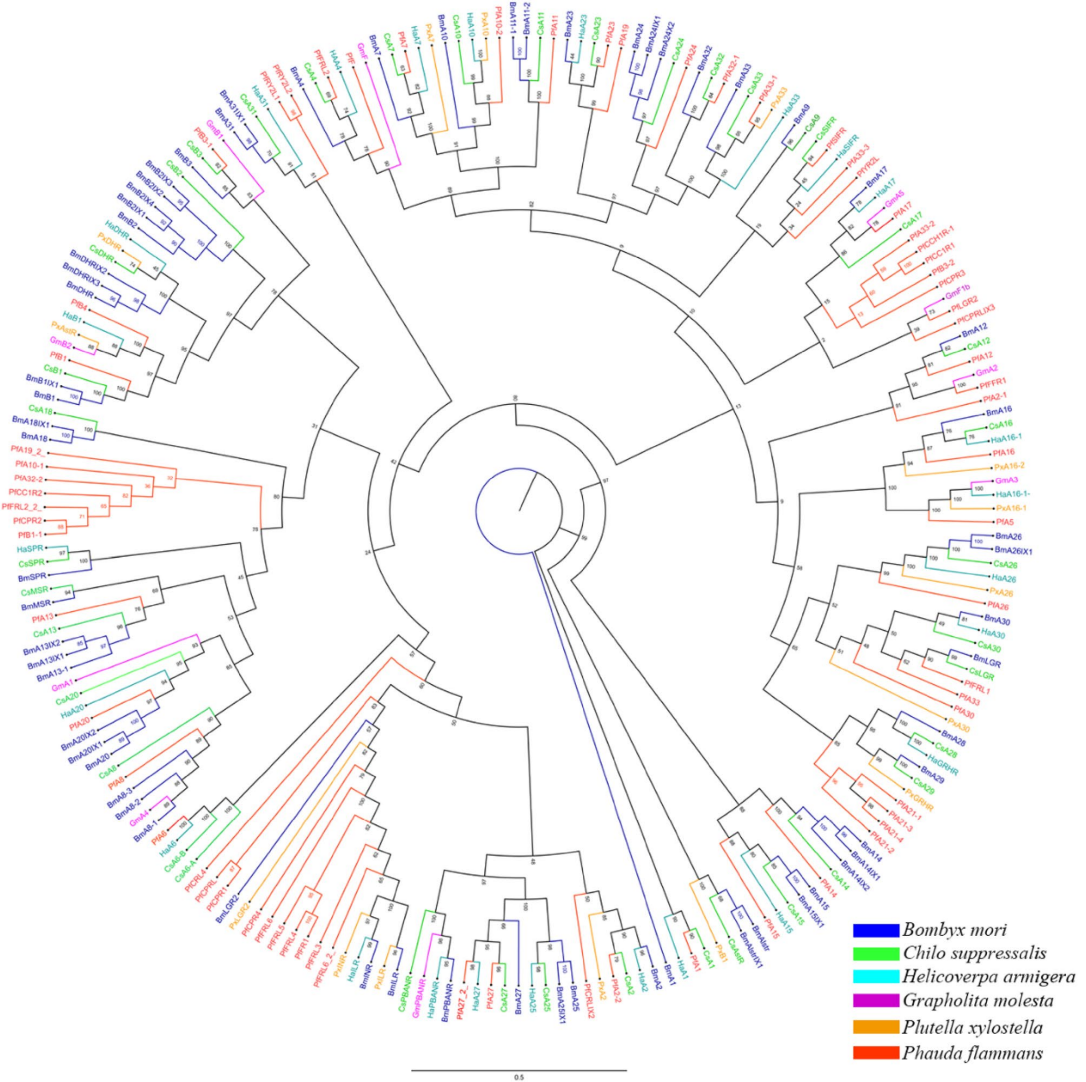
Phylogenetic tree analysis of lepidopterous neuropeptide receptors. Bm: *B. mori*; Cs: *C. suppressalis*; Ha: *H. armigera*; Gm: *G. molesta*; Pf: *P. flammans*, Px: *Pl. xylostella*. The *P. flammans* neuropeptide receptors are labeled with red, and the colors of other species are shown in the icon. The tree was conducted with MEGA 7.0, using the Maximum-Likelihood method and the bootstrap analysis with 1000 replicates.

The 152 reported neuropeptide receptor sequences of *B. mori*, *C. suppressalis*, *H. armigera*, *G. molesta* and *Pl. xylostella* from lepidopteran and the identified neuropeptide receptors of *P. flammans* were used to construct an interspecies phylogenetic tree (Fig. 5). The results showed that A26 of *P. flammans* was clustered together with the A26 of *B. mori*, *C. suppressalis* and *Pl. xylostella* in 100; B3-1 of *P. flammans* was clustered together with the B3 of *B. mori* and *C. suppressalis*; A6-b of *P. flammans* was clustered together with the A6 of *H. armigera*. A19-2-, A10-1, A32-2, CCH1R2, B3-2 and FRL2 were individually clustered together. A21-1, A21-2, A21-3 and A21-4 were individually clustered together, and it’s the same with CCHIR-1, CC1R1, CPR2 and CPR3. It showed that neuropeptide receptor emerged highly differentiation in *P. flammans*. The remaining receptors were clustered together with the orthologs from other lepidopteran insects in the same clade.

### Tissue expression profile in female and male adults

**Figure 6:**
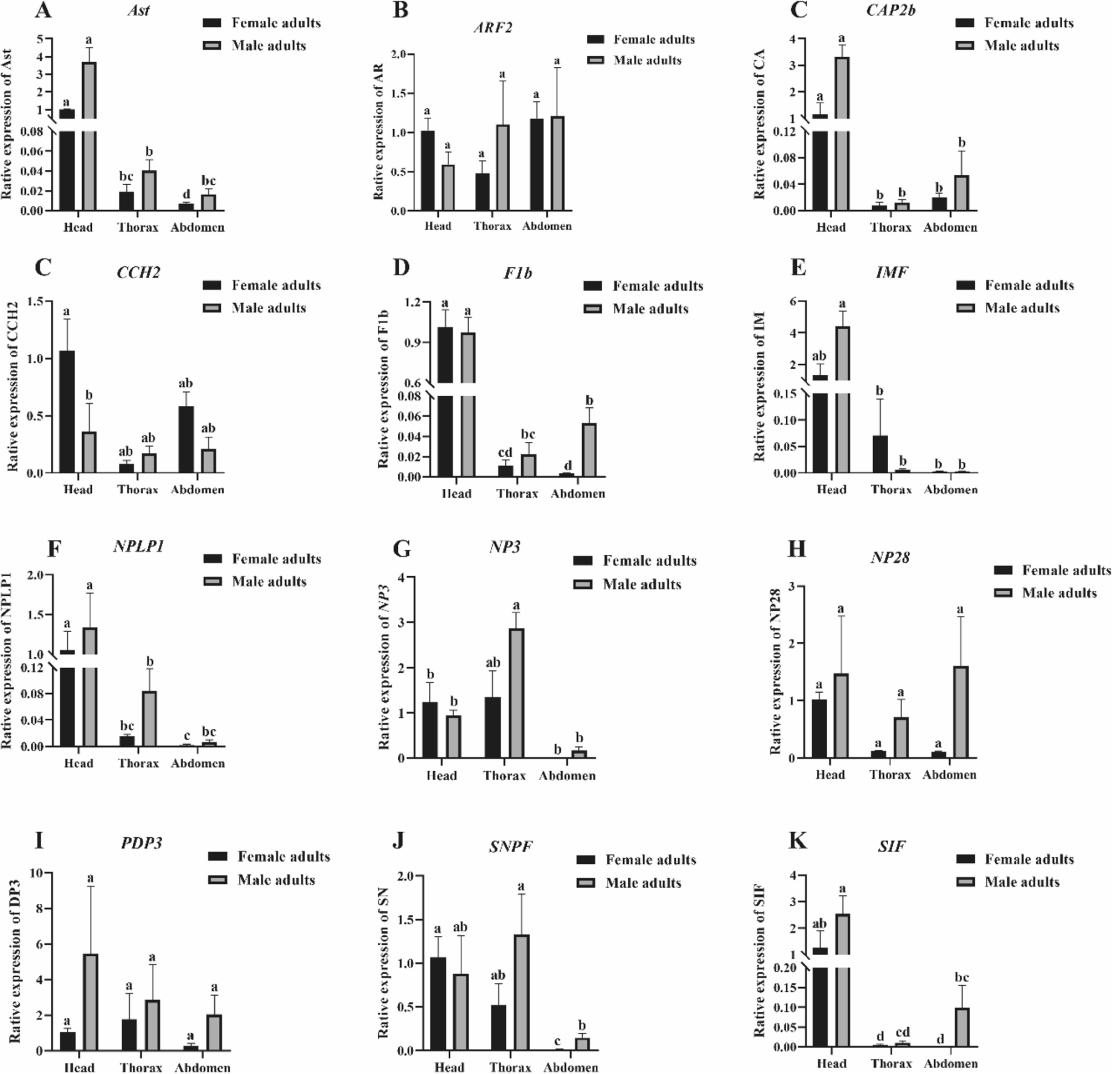
Tissue expression of neuropeptides in both sexes of *P. flammans*. Data are expressed as mean ± standard error (SE). Values followed by different letters are significant (*P* < 0.05) analyzed by Tukey’s honestly significant difference (HSD) multiple test.

The expression profiles of 12 neuropeptides of *P. flammans* in heads, thoraxes, and abdomens of male and female adults were showed in Fig. 6. The expression of *CA*, *LM*, *Ast*, *F1b*, and *NPLP1* were significantly higher in heads than other two body parts in both female and male. While the expression level of *AR*, *DP3*, and *NP28* showed no significant difference in these three body parts in both sexes. All these neuropeptides showed no difference in female and male heads except for *CCH2*.

## Discussion

Neuropeptides and receptors regulate a wide range of physiological processes in insects. Transcriptome sequencing is fundamental to dentification of genes, and identification of neuropeptides and their receptors is the first and foremost step of deep function depth studies in physiological processes. However, the types and expression of neuropeptides and their receptors in *P. flammans* are unavailable. Therefore, a sequencing analysis was performed of head in *P. flammans*. After high-throughput sequencing, among the 99,386 unigene acquired by the assembly program Trinity, 40.38% could be annotated through NR, KEGG, Swiss-Prot, KOG and GO databases, implies that not all unigene contain annotated genes. Some unigene may be non-coding which do not BLAST with the non-redundant protein/nucleotide database. Compared with the transcriptome data of head from *Mythimna separata*^43^ and *H. armigera*^44^, the *P. flammans* had a similar result. Q20 and Q30 values were all > 93%, and GC content was similar, indicating that the data was accurate and reliable. In Nr databases, the number of homologous sequences most with *P. flammans* include *E. japonica*, *B. mori*, *G. mellonella*, which all order of Lepidoptera, suggesting that the transcriptome was commendably sequenced and annotated. Overall, the assembly quality of transcriptome was adequate.

Basically, the number of achieved target gene should be closely related to the sample resource and expression abundance in addition to sequencing depth with species specificity. The same was true for neuropeptides and neuropeptide receptors in *P. flammans*. Totally, 15 neuropeptides and 66 neuropeptide receptors were identified from head of adult *P. flammans*, which was different with other lepidopteran species^44–47^ and should partly be relevant with their differences in sample physiological status. For example, in *B. mori*, 32 neuropeptide genes and 6 neuropeptide-like precursor genes were identified from larval and pupal brain^45^. In *C. suppressalis*, 43 neuropeptide precursors and 51 putative neuropeptide G protein-coupled receptors were identified the fifth instar larval central nervous system including brain, suboeophageal ganglion, thoracic ganglion, and the abdominal ganglion^46^. In *H. armigera*, 34 neuropeptides and peptide hormones, 17 neurotransmitter precursor processing enzymes, and 58 neurotransmitter receptors were identified from mixed pupa and adult head^44^. It seems that more sophisticated sampling would yield a larger number of neuropeptides and receptor genes. In addition, the number of identified genes might also have species specificity. The number of identified neuropeptides of *P. flammans* was less than the number of some other lepidopteran species such as from the transcriptome data of head, such as *A. hetaohei*^47^.

There are several factors that may account for the difference in the number of identified genes of specific functions which has been discussed^48,49^. Firstly, the head used as the sequenced samples did not cover complete the individual and all stages of life cycle. Secondly, some genes with small expression levels made it impossible to quantitatively measure the gene expressions in samples presented a not expression state, or them may not have been expressed at all. And then, due to does not involve the modification of corresponding protein-coding regions, many genes lack strong sequence conservation, their clear orthologs could not be found in *P. flammans* based on homology searches. The neuropeptides may truly present with small quantity in *P. flammans* because of highly species specificity which needs further investigation.

In this analysis, female and male head transcriptome in *P. flammans* was performed with focus on the feeding behavior regulation and sexual difference. Only a total of 217 differentially expressed genes were screened, with 156 genes up-regulated and 61 genes down-regulated. Approximately 12% of these DGEs were olfactory association related genes (Supporting information 1), while no neuropeptide or neuropeptide receptor were found. Moreover, some neuropeptide and neuropeptide receptors have reported to induce sex pheromone biosynthesis and feeding behaviors^50,51^. Therefore, the small number of neuropeptides and neuropeptide receptors from head in *P. flammans* might lead to those gene tightly to olfactory regulation and reduce workload in targeting behavior regulation gene. For instance, there were 19 unigenes which located in the Ko00981, the insect hormone biosynthesis pathway, where only unigene0063695 and unigene0024395 were significantly differential expressed and annotated as gene cytochrome P450 18a1 (*CYP18A1*) and farnesol dehydrogenase-like (*FoLDH*), respectively (Fig. S1). CYP18A1 played a controlling role in 20-hydroxyecdysone inactivation in *B. mori*^52^, and were reported to function in development, especially to regulate dimorphic metamorphosis via by insect hormones^53,54^. FoLDH could induce oxidation of farnesol to farnesal and produce the second branch of JH III in *Pl. xylostella*^55^. In addition, DGE Unigene0010507 was annotated as juvenile hormone binding-like protein (Supporting information 1) and how the relationship between it and insect hormone biosynthesis pathway attracted our attention. Therefore, the functions of these DGEs require further analysis and validation in *P. flammans*.

Neuropeptides and neuropeptide receptors identified from the head of *P. flammans* showed no significant difference between male and female adults, however, they are crucial in regulating a range of physiological functions, including development, reproduction and feeding^56^. Therefore, identification and analysis neuropeptides and their receptors are still necessary and meaningful. In the aspects of feeding behavior, for example, short neuropeptide F peptide is expressed in the nervous system and it regulates food intake and body size by overexpression of SNPF with regulate expression of insulin-like peptides in *Drosophila*^57^. Another example, NPF as a pleiotropic factor, is well known for its role in the regulation of feeding^58^, through activating neuropeptide G protein-coupled receptor to regulate feeding and growth in *B. mori*^59,60^, which is also a daily oligophagous species that might provide some references for *P. flammans*. In the aspects of sexual difference, the release of SIFamide in the brain could inhibit sexual behavior until the flies encounter the right physiological conditions^61^, which might also function in sexual differences. All these deductions need further confirmation far and away via quantitative PCR, tissue localization, function inhibition and so on.

Neuropeptides were less abundant in this study and easier to target their expression in different tissues. From a general view, all the measured neuropeptides were expressed highly or moderately in heads where they were identified from transcriptome (Fig. 6). As mentioned above, the neuropeptide *CCH2* and the neuropeptide receptor CCH1R-1 could be identified, but them were no significantly different expressed in the head of females and males (Supporting information 1), while quantitative PCR results showed a slightly significant difference in *CHH2* (Fig. 6C). Similar results were also found in *CCHamide 1* and *CCHamide 2* which were significantly different expressed in the head of females and males of *A. hetaohei*^47^. Fold changes in *CHH2* expression in female and male heads by QPCR was minor, and therefore the conflicting point shall result from the sensitiveness of QPCR and RNA-Seq methods. In addition, SIFamide a highly conserved neuropeptide and has been reported to modulate courtship behavior differently in female and male *Drosophila*^61,62^, which making *SIF* a gene of interest in *P. flammans*.

The drawbacks of the adopted second generation sequencing were undoubted. However, we did obtain a mass of valuable genetic data for *P. flammans* with a tight fund, especially in neuropeptides and their receptors. Novel neuropeptides could be supplemented via Genomics- and peptidomics -based discovery in the future^63^. Moreover, association of multiple omics, such as full-length transcriptome, proteome and metabolome might be needed^64^, which would contribute to the feeding and sexual behavior regulation researches in this diurnal moth *P. flammans* by outlining a chain with cascaded neuropeptide, neuropeptide receptor, pheromone metabolism and behavior.

## Conclusion

In this study, 15 neuropeptides and 66 neuropeptide receptors were identified from *P. flammans*, and the genes exhibited no significantly different expression in head between female and male. Phylogenetic analyses tree with neuropeptides and receptors of other lepidopteran species illustrated clear interspecies relationships and contributed to further function understanding. Our findings enriched neuropeptides and neuropeptide receptor gene database, which provide a theoretical support for pest management strategies and physiological and biochemical researches in *P. flammans*.

## Supplementary Information

Supplementary Information 1.

Supplementary Information 2.

Supplementary Information 3.
