# Supplementary figures and images for: Identification of neuropeptides and neuropeptide receptor genes in Phauda flammans (Walker)
Source: Sci Rep. 2022 Jun 14;12:9892. doi: 10.1038/s41598-022-13590-7 (PMC9198061; doi:10.1038/s41598-022-13590-7)

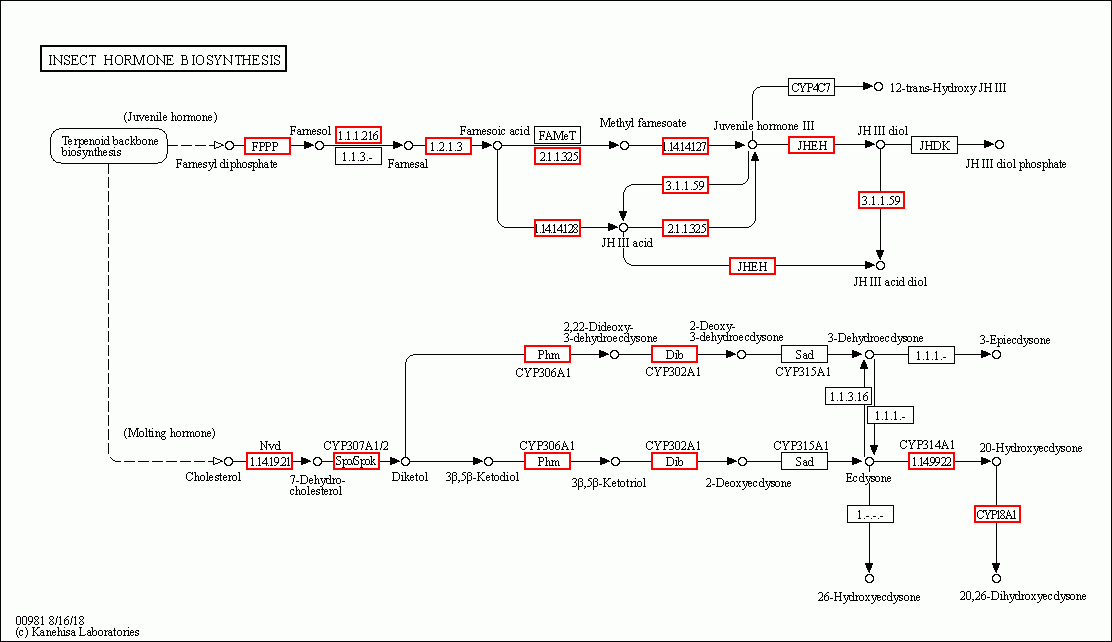

Supplement: Supplementary file 1 — Supplementary Information 1. [file 41598_2022_13590_MOESM1_ESM.png]
