# Supplementary material for: Identification of neuropeptides and neuropeptide receptor genes in Phauda flammans (Walker)
Source: Sci Rep. 2022 Jun 14;12:9892. doi: 10.1038/s41598-022-13590-7 (PMC9198061; doi:10.1038/s41598-022-13590-7)
Supplement: Supplementary file 3 — Supplementary Information 3. [file 41598_2022_13590_MOESM3_ESM.docx]

Table S1 Primers used during qPCR analysis in *Phauda flammans*

| Accession No. | Primer name | Sequence (5´ - 3´) | Amplicon product （bp） |
| --- | --- | --- | --- |
|  | *Ast*-F | GCAGCTTGAAAGTGAAGATCG | 149 |
|  | *Ast*-R | AATAGCACTGTCGGAATCGG |  |
|  | *AR*-F | TGCTAAGAAACATGAACCAATCAG | 195 |
|  | *AR*-R | ACACATTTTCATTATCTGGCTTGG |  |
|  | *CA*-F | AGAACTCCAGACTCCACTCAG | 189 |
|  | *CA*-R | ATTAGTTCTGGCTCGCTGTG |  |
|  | *CCH2*-F | TTTGGTCACTCGTGCTATGG | 160 |
|  | *CCH2*-R | TGTCATCTCCTGGAAGTTGC |  |
|  | *F1b*-F | CTGCTAGACCAAGGATTGACC | 123 |
|  | *F1b*-R | TGTGGTAGGAGTAAATGCGG |  |
|  | *IM*-F | TGTGGCAATACTCCTATCTGTTG | 110 |
|  | *IM*-R | CTCGTGGGTCATAATCTGATGG |  |
|  | *NPLP1*-F | TTTAGGTTTACAGGCGGACG | 138 |
|  | *NPLP1*-R | TGGTTCAGTTTCGGCACTAG |  |
|  | *NP3*-F | CATGGCATTTATGGAGCACC | 98 |
|  | *NP3*-R | GTAGCGATCCTGACAGTGTTG |  |
|  | *NP28*-F | GATGGGTGGATATGGTTATGGG | 91 |
|  | *NP28*-R | AACGTTGGCTGTGGAGATAG |  |
|  | *DP3*-F | TGGGCTTCGTACTGGTTTG | 178 |
|  | *DP3*-R | GTAATTCGAGAGTCTGGTCCG |  |
|  | *SN*-F | TGGACTGTATTCGTCATTGGG | 152 |
|  | *SN*-R | GTAGTTCGTCTTCATCGCTAGG |  |
|  | *SIF*-F | TGTCATTTCACTTTGCTTCATCG | 166 |
|  | *SIF*-R | CATGGTAATTTCACAGAGCGC |  |
| MN852477 | *TUB1*-F | GATGCCGACCGACAAGACTA | 155 |
|  | *TUB1*-R | ACAACTGCCTGTATGTGCCA |  |
| MN852481 | *GAPDH*-F | AACTGCCTTGCTCCACTAGC | 145 |
|  | *GAPDH*-R | GAGCACCACGACCA TCTCTC |  |
